# Evaluation of Fenton oxidation process coupled with biological treatment for the removal of reactive black 5 from aqueous solution

**DOI:** 10.1186/2052-336X-11-13

**Published:** 2013-06-28

**Authors:** Pegah Bahmani, Roshanak Rezaei Kalantary, Ali Esrafili, Mitra Gholami, Ahmad Jonidi Jafari

**Affiliations:** 1 Department of Environmental Health Engineering, School of Public Health, Tehran University of Medical Sciences, Tehran, Iran; 2 Center for Water Quality Research (CWQR), Institute for Environmental Research (IER), Tehran University of Medical Sciences, Tehran, Iran; 3 Department of Environmental Health Engineering, School of Medical Sciences, TarbiatModaresUniversity, Tehran, Iran

**Keywords:** Fenton oxidation process, Anoxic biological treatment, Decolorization, Mineralization, Azo dyes

## Abstract

Biodegradation of azo dyes is difficult due to their complex structures and low BOD to COD ratios. In the present study, the efficiency of using Fenton’s reagent (H_2_O_2_ + Fe^2+^) as a pretreatment process to enhance microbial transformation of reactive black 5 (RB5) in an aqueous system was evaluated. The RB5 with an initial concentration of 250 mg/L was decolorized up to 90% in 60 h by using a bacterial consortium. Fenton’s reagent at a Fe^2+^ concentration of 0.5 mM and H_2_O_2_ concentration of 2.9 mM (molar ratio, 1:5.8) was most effective for decolorization at pH = 3.0. The extent of RB5 removal by the combined Fenton–biotreatment was about 2 times higher than that of biotreatment alone. The production of some aromatic amines intermediates implied partial mineralization of the RB5 in Fenton treatment alone; in addition, decreasing of GC-MS peaks suggested that dearomatization occurred in Fenton-biological process. Fenton pretreatment seems to be a cost–effective option for the biotreatment of azo dyes, due mainly to the lower doses of chemicals, lower sludge generation, and saving of time. Our results demonstrated positive effects of inoculating bacterial consortium which was capable of dye biodegradation with a Fenton’s pretreatment step as well as the benefits of low time required for the biological process. In addition, the potential of field performance of Fenton-biological process because of using bacterial consortium is an other positive effect of it.

## Introduction

Textile industries produce large amounts of liquid effluents. More than 7 × 10^5^ tones of synthetic dyes are produced annually by textile industries [1,2], of which 2.8 × 10^5^ tones are discharged [3-5]. Azo dyes belong to the largest and most versatile class of dyes [6] and present about 60-70% of dye stuff released into the environment [7]. Discharge of wastewater containing azo dyes results in the pollution of aquatic systems and, therefore, causes adverse effects on human health [8]. Generally, most of these dyes or their cleavage products (i.e., aromatic amines) may be mutagen or carcinogen [9-11]. Hence, treatment of wastewater containing such dyes is essential to prevent deterioration of ecosystems [12,13].

Previous studies have shown that the physicochemical methods of treatment (i.e., adsorption, ozonation, and advanced photochemical oxidation) have been applied for the treatment of the effluents [14-17]. However, these processes have achieved limited success because they are associated with the generation of large amounts of sludge, which is difficult to dispose [18], or may cause secondary pollution due to the use of excess chemical materials [13]. Although biological treatment is a well-established, cost-effective technology, it has been proven inefficient for the degradation of azo dyes like Reactive Black 5 (RB5). This is due primarily to the fact that they have nitrogen to nitrogen double bonds (-N = N-), aromatic rings, and low BOD to COD ratios (<0.1) [19].

Due to the combination of both environmental and economic advantages, the coupling between chemical and biological processes can be a suitable solution for the removal of toxic compounds from aqueous medium [20-22]. Advanced oxidation processes (AOPs) such as Fenton reagents (H_2_O_2_/Fe^2+^) may improve the biodegradability of synthetic dyes through the use of short–lived, highly reactive hydroxyl radicals (OH•) [23,24]. Moreover, Fenton oxidant is commercially available and easy to handle environmentally. In most cases, Fenton is used for both decolorization and mineralization simultaneously; thus higher doses of H_2_O_2_ and Fe^2+^ ions are required. However, smaller amounts of Fenton reagents are required when combined with biological processes. Lucas et al. (2007) showed that *C. Oleophila* as a pure culture had a good efficiency in degradation of the dye after Fenton process [25]. Decolorization of Reactive Red 2 by *Pseudomonas* sp. SUK1 showed that the acclimated pure bacteria have better efficiency for dye removal than an acclimated consortium of the bacteria [18]. Usually the effect of pure culture is more than that of mixed culture; however, since the maintenance of pure culture in the environment is not easy, the utilization of a consortium of the microorganism is preferable [1]. Mohana et al. (2008) used a novel bacterial consortium for decolorization of Direct Black 22 [12].

In this study, the Fenton oxidation process was used prior to biodegradation for breaking the unsaturated bonds (-N = N-) of RB5. Furthermore, degradation can be achieved by a consortium of acclimated bacteria. The combined Fenton oxidation/bioreactor process is expected to be more economical and effective compared to any single treatment technology for the treatment of RB5. Because of using bacterial consortium which had not been used before in such coupled process, the Fenton-biological process has the potential of field performance.

Therefore, the aim of the present study was to investigate the Fenton oxidation process to achieve complete decolorization of RB5 and partial cleavage of aromatic amines to make them easily biodegradable by a bacterial consortium.

## Material and methods

### Chemicals

**Figure 1:**
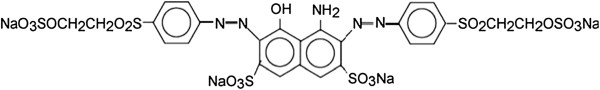
Chemical structure of the azo dye used in this study.

Commercial grade RB5 (as a model dye) was purchased from Alvan Sabet Company (Hamedan, Iran) and used without any further purification. Figure 1 shows the structure of RB5. A stock solution of the dye (1000 mg/L) was prepared in distilled water and used for all experiments. Dichloromethane, acetone, and methanol were supplied in HPLC grade by Merck, Germany. Analytical grade chemicals used for mineral salt medium (MSM) as well as culture media were purchased from Merck Company.

Ferrous sulfate hepta hydrate (FeSO_4_ · 7H_2_O) was used as the source of Fe^2+^ catalyst in the Fenton process. Hydrogen peroxide solution (30%, w/w), sulfuric acid, and sodium hydroxide were also purchased from Merck Company. All of these chemicals were of analytical grade.

### Methods

The process applied in the present work consisted of three main phases: biological treatment; advanced oxidation process (i.e. Fenton oxidation process); and Fenton-biological process, which itself consisted of two stages, including advanced oxidation process as stage I and biological treatment as stage II.

### Screening of dye degrader consortia

The source of acclimated microorganisms was activated sludge from the secondary sedimentation basin of Nima Nassag Wastewater Treatment Plant, Tehran, Iran.

The mineral salt medium (MSM) contained the following (per liter): 6 g NaH_2_PO_4_, 3 g KH_2_PO_4_, 1 g NH_4_Cl, 0.5 g NaCl, and 1 mL 1 M MgSO_4_, all of which were of analytical grade. About 10 g of the sludge was added to 100 mL of MSM and put on the shaker at 150 RPM at 37°C for 12 h. After allowing the sludge to settle down for 2 h, the supernatant was inoculated to Luria Broth (LB) agar medium which contained the following (per liter): 10 g Casein enzymic hydrolyzate, 5 g yeast extract, 5 g NaCl, 15 g agar, and 10 mg RB5. The capable microorganisms of degrading dye were picked up on the basis of their ability to form clear zones on these plates. The colonies were transferred to LB agar slant for following experiments and identification. The isolated bacteria were detected on the basis of morphological, physiological, and biochemical characteristics according to Winn et. al (2006) [26].

### Biological treatment

Experiments were performed in a 250 mL glass beaker containing 200 mL of LB media inoculated with a consortium of bacteria with an optical density of 0.1 at 600 nm. Different concentrations of RB5 dye (50, 250, and 500 mg/L) were added to the LB medium. To investigate the non–biological removal, the samples had similar blanks which were free of inoculation. In addition, to investigate the adsorption by bacterial cell, killed controls were used too. A dye-free microbial blank was used to survey the changes in microbial population. All the reactors were incubated at 30 ± 2°C in dark static conditions. At different time intervals, the samples were drawn and centrifuged at 6000 RPM for 15 min, and then the dye concentration was determined by a spectrophotometer at 600 nm [27].

### Optimization studies for decolorization of dye using Fenton reagent

Optimization studies were carried out to optimize the time and low doses of H_2_O_2_ and Fe^2+^ ions for the decolorization of different concentrations of the RB5 dye. During the optimization for pH and time, doses of both H_2_O_2_ and Fe^2+^ ions were kept constant while pH and time were changed in the range of 2–8 and 15–60 min, respectively. The experiments were then conducted in a 250 mL glass beaker at the optimum time and pH applying different doses of H_2_O_2_ (i.e. 0.73, 1.47, 2.2, 2.9, and 3.68 mM) and Fe^2+^ (i.e. 0.1, 0.5, 1, and 2 mM) in 20 trials. The experiments were carried out in triplicate.

Dye solution was prepared by diluting the stock solution in distilled water, and the pH was adjusted by using 0.5 M H_2_SO_4_. A measured amount of Fe^2+^ ions and H_2_O_2_ were added to the reactor, and the reaction was allowed to continue for 15 min on an orbital shaker (200 RPM) at ambient temperature (22–25°C). Afterwards, the supernatant was analyzed for color removal. In order to prevent any photo degradation, all the reactors were covered with foil.

### Fenton-biological treatment

In stage I of the Fenton-biological process two levels of response were selected: high level (for the best removal) and low level (only for Fenton pretreatment and allowing for biological reaction). The first stage was similar to the Fenton oxidation process alone. Following the Fenton pretreatment, in stage II, pH of the sample was adjusted to 7 by using 1 M NaOH [28,29]. Then, it was inoculated with a consortium of bacteria in LB with an optical density of 0.1 at 600 nm and kept at 30 ± 2°C in dark condition; the samples were then drawn at different time intervals. All the experiments were carried out in triplicate.

### Analytical methods

A CECIL-model 7100 UV/vis spectrophotometer was used for monitoring the decolorization at λ_max_ = 600 nm for RB5 [30]. The remained RB5 and its metabolites in the liquid was extracted with dichloromethane, which was then centrifuged (Hettich D7200) for 10 min at 6000 RPM. Degradation products were identified by comparing the retention time and fragmentation pattern, as well as based on the spectra in the GC-MS software.

The gas chromatography instrument comprised an Agilent (Centerville Road, Wilmington, USA) 7890A GC coupled with an Agilent MSD 5975C quadrupole mass spectrometer. The GC was fitted with HP-5 MS capillary column (30 m × 0.25 mm i.d., 0.25 μm film thickness) from Agilent J&W Scientific (Folsom, CA, USA). Helium (99.999%) was used as the carrier gas at 1.0 ml min^-1^. The following temperature program was employed: 50°C for 1 min, increased to 280°C at 10°C min^-1^, and held for 5 min. The MS quadruple and the MS source temperatures were set at 150 and 230°C, respectively. Data acquisition was performed in the full scan mode (m/z in the range of 50–550).

## Results

### Decolorization of different concentrations of dyes in biological treatment

**Figure 2:**
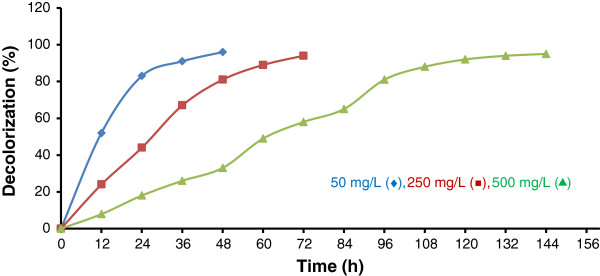
The percentage of RB5 decolorization at various initial dye concentrationsin the biological process.

The bacterial consortium was chosen on the basis of its ability to form a clear zone on the plate containing LB agar and RB5. To assess the maximum decolorization ability of the consortium, it was tested against different concentrations of the dye (Figure 2).

At a dye concentration of 50 mg/L, more than 90% dye removal was achieved within 36 h; similar decolorization was also observed at concentrations of 250 and 500 mg/L within 60 and 108 h, respectively. The time required for complete decolorization of 50 mg/L of the dye was 48 h, whereas it was 72 h for a dye concentration of 250 mg/L. As concentration increased to 500 mg/L, the time required for decolorization increased to 168 h. Less than 3% of the removal was related to the adsorption of dye on the cell mass (data are not shown).

### Decolorization of RB5 in Fenton process

**Figure 3:**
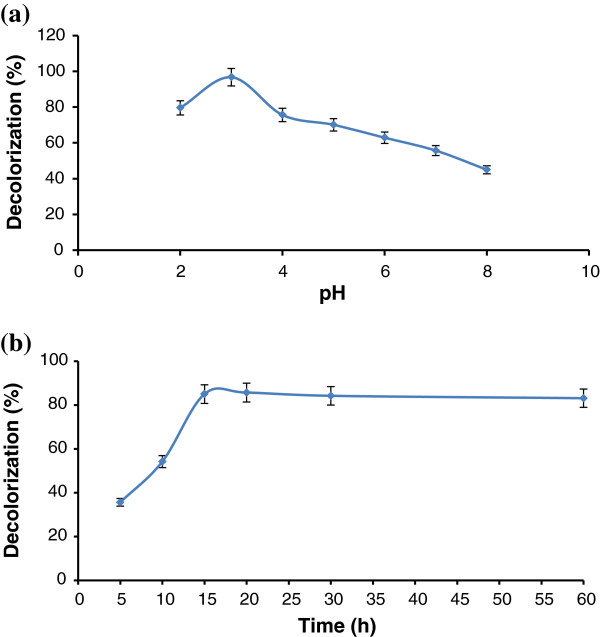
**Effects of (a) initial pH and (b) reaction time on RB5 decolorization in the Fenton process (reaction conditions: initial dye concentration of 250 mg/L; H**_**2**_**O**_**2**_**dose of 2.9 mM, and Fe**^**2+**^**does of 0.5 mM).**

**Figure 4:**
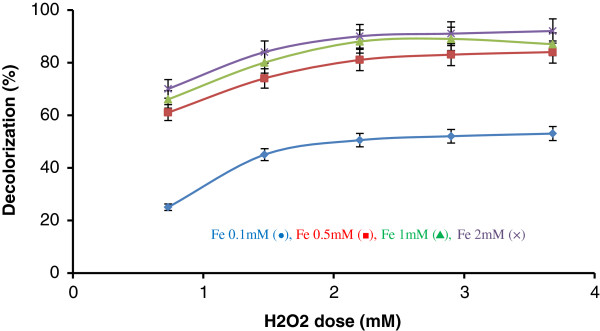
**Effect of different concentrations of H**_**2**_**O**_**2 **_**and Fe **^**2+ **^**on RB5 decolorization in the Fenton process (experimental conditions: [RB5] = 250 mg/L; pH = 3; reaction time = 15 min).**

All the experiments were carried out at a dye concentration of 250 mg/L. The influence of initial pH and time on the effectiveness of Fenton processes for RB5 degradation (expressed as color removal efficiency) is shown in Figure 3(a) and (b), respectively. The maximum dye removal was achieved at pH 3 in 15 min; hence, the initial pH was kept constant at 3 for all other Fenton experiments, and the reactions were done in 15 min. Figure 4 demonstrates the effect of iron and H_2_O_2_ concentrations on the dye removal. The RB5 removal increased by increasing concentrations Fenton reagents. Increasing of dye removal was significant by increasing Fe^2+^ from 0.1 to 0.5 mM.

### Decolorization of RB5 in combined process

**Figure 5:**
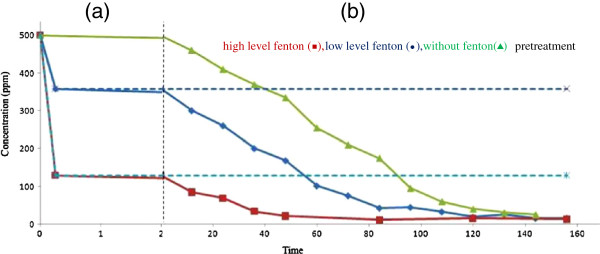
**Biotransformation profile of RB5 treated with the combined Fenton–biological process; Fenton reagents with Fe**^**2+**^**: H**_**2**_**O**_**2 **_**molar ratio of 0.5:2.9 (a) in first 2 h; (b) after 2 h.**

The time–course for the transformation of Fenton pretreated RB5 (500 mg/L) by the bacterial consortium is shown in Figure 5. The overall removal of more than 90% of RB5 was achieved in 36, 84, and 108 h for high and low levels of Fenton reagents and without pretreatment, respectively. Application of the Fenton pretreatment led to removal efficiencies of 28% and 74% for low and high levels of it, respectively.

## Discussion

### Biological treatment

The bacterial consortium, consisted of *Alcaligenes Xylosoxidans* and *Alcaligenes Denitrificans*, could completely decolorize RB5 when Casein enzymic hydrolysate and yeast extract were present in the medium. In the absence of yeast extract and Casein enzymic hydrolysate, the consortium did not have the ability to decolorize RB5. Moosvi et al. (2005) reported that an isolated bacterial consortium RVM 11.1 did not show decolorization when yeast extracts and glucose were omitted from the medium [31].

The decolorizing potential of the bacterial consortium was studied using RB5 at different initial concentrations in the range of 50–500 mg/L. The rate of decolorization increased with an increase in the initial dye concentration. Similar observations have also been reported earlier for decolorization of RVM 11.1 up to 200 mg/L by Moosvi et al. (2005). However, they reported that further increase in the dye concentration resulted in the reduction of decolorization rates, while this led to a further increase of decolorization rates in our work [31]. The rate of RB5 removal by the bacterial consortium was about 0.9, 2.1, and 3.1 mg/L.h for initial concentrations of 50, 250, and 500 mg/L, respectively. Azo dyes generally contain one or more sulphonic–acid groups on aromatic rings, which might inhibit the growth of microorganisms and result in less biodegradation [3,32]; but using a mix culture of bacteria may result in higher levels of biodegradation, mainly because of their potential to overcome on decrease in their density. On the other hand, in the environment, the performance of dye removal by using a pure culture is not possible [1].

The time required for complete decolorization at a dye concentration of 50 mg/L was 60 h, which was similar to that reported by Kalme et al. (2007). However, the time required for complete decolorization at dye concentrations of 250 and 500 mg/L was 4 and 7 days, respectively; this is better than the results observed by Kalme et al. (2007) [33]. These times required for biodegradation suggest that an acceptably high level of color removal cannot be achieved by biodegradation alone.

### Fenton treatment

The effect of pH on the decolorization of RB5 by Fenton process is shown in Figure 3a. The experiments were carried out at pH values between 2 and 8. The optimal pH values were obtained at pH 3 with the highest decolorization of dye, which usually falls in the acidic range of pH in Fenton processes [34,35]. Under more alkaline conditions (pH > 4), the precipitation of iron hydroxide occurs and the concentration of dissolved Fe^3+^ and, consequently, Fe^2+^ decreases. On the other hand, in this condition hydrogen peroxide is unstable and fewer hydroxyl radicals are formed, which may reduce the efficiency of the process [34]. It is noteworthy that for pH values below 3, the reduction in the production of hydroxyl radical occurs too [35].

Figure 3b indicated that the maximum dye removal was achieved at 15 min and color removal remained unaffected for the reaction times between 30 and 120 min. High percents of dye removal were also obtained by Rodrigues et al. (2009) and Hsing et al. (2007) in less than 20 min [34,36].

In subsequent runs, consequently, the reaction time and the initial pH were kept constant at 15 min and 3, respectively. The concentration of hydrogen peroxide was the controlling factor for RB5 decolorization, which was consumed in the early stages of the reaction. As the H_2_O_2_ dosage was increased from 0.73 to 2.9 mM, the efficiency of RB5 decolorization increased up to 20–25% at 15 min of the reaction time. This indicates that the observed increase in the efficiency of RB5 decolorization was due to the increase in OH• concentration by the addition of H_2_O_2_, as seen in Figure 4. However, further increase in hydrogen peroxide concentration, H_2_O_2_ > 2.9 mM,did not affect the degradation efficiency, which can be attributed to the scavenging effect of hydroxyl radical, according to Eq. 1 and 2:

(1) $$HO^{•}+H_{2}O_{2}\to H_{2}O+HO_{2}^{•}$$

(2) $$HO_{2}^{•}+HO^{•}\to H_{2}O+O_{2}$$

For different concentrations of different dyes, the optimum dose of H_2_O_2_ for dye degradation has been reported to be in the range of 0.73–72 mM by Tantak et al. (2006), Lucas et al. (2006) and Hsing et al. (2007) [4,35,36].

Degradation efficiency of dye was also influenced by the concentration of Fe^2+^ ions which catalyze hydrogen peroxide decomposition, resulting in the production of OH radicals and, consequently, degradation of the dye. With increasing doses of ferrous ions from 0.1 to 0.5 mM, the degradation efficiency of RB5 also increased up to 91.5%. From this point forward, however, further addition of iron became inefficient. It can be seen from the results presented in Figure 4 that higher RB5 degradation levels were obtained with a lower Fe^2+^ concentration (0.5 mM). Hsuing et al. (2007) reported a required Fe^2+^dose of 8.93 mM for the removal of Acid Orange 6 [36].

The optimal Fenton reagent ratio for RB5 degradation established on the basis of dye removal efficiency is 0.5 mM Fe^2+^: 2.9 mM H_2_O_2_ at pH 3, where the maximum dye removal efficiency of 91.5% was achieved. According to the previous studies, typical values of Fe^2+^/H_2_O_2_ are in the range of 1:5 to 1:25 [30]. Based on the results from the present work, dosesof 2.9 mM of H_2_O_2_ and 0.5 mM of Fe^2+^were selected for conducting dye removal experiments by the combined Fenton–biotreatment process.

### Combining Fenton’s reagent and biological treatment

In this study, two processes, i.e. Fenton’s reagents as a pretreatment followed by biological treatment including a bacterial consortium, were integrated for optimization purposes.

The major purpose of this integrated process was to reduce the treatment costs, particularly the hydrogen peroxide concentration used in the Fenton process for decolorizing a RB5 concentration of 500 mg/L. It is noteworthy that during the Fenton pretreatment process, the reaction can also be carried out at a low ferrous ion dose; therefore, the amount of sludge generation would be negligible. This can solve the problem of sludge disposal, thereby adding to the cost–effectiveness of the process [19].

Two levels of high and low dose of Fenton’s reagents of second phase results were selected for first stage. Then, the biodegradation of remaining RB5 and its metabolites were conducted by inoculating the bacterial consortium and incubating in static condition. At lower dose of Fenton’s reagent the negative effect of H_2_O_2_ residual on the biological stage was minimized [19], mainly because of fast consumption of the oxidant [4,37-39]; on the other hand, there is no need for separating the precipitated iron oxides [40]. High dose of Fenton’s reagent was used for the highest effect on color removal.

Results indicated that a significant reduction in the biological detention time was achieved (Figure 5). Biological treatment as a single process could achieve an acceptable reduction at an initial dye concentration of 500 mg/L after about 6 days. However, in the combined Fenton–biotreatment system could achieve a removal efficiency of about 90% in almost 3 days, which is considered to be more cost–effective because shorter HRTs decrease the installation costs.

**Figure 6:**
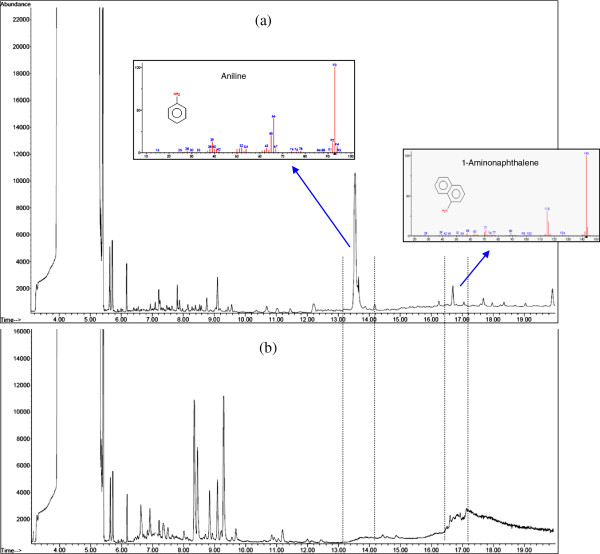
GC-MS chromatograms of RB5 decomposition products in the Fenton–biological process; (a) after Fenton stage, (b) after biological stage.

The GC–MS spectrum showed various peaks indicating the partial mineralization of RB5 after the Fenton process (Figure 6a). The retention time of peaks and suggested MW indicated that in the Fenton process, RB5 was most likely broken down into compounds with lower MW.It also suggested that decolorization was related to the breaking of azo bonds which are associated with chromophores, i.e. conjugated unsaturated bonds (–N = N–) in the molecule [15,41]. In this stage, some aromatic amines intermediates, such as Aniline and 1-Aminonaphthalene, may have been produced, indicating partial mineralization of RB5. The production of aromatic amines is in agreement with the results from the study of Kang et al. (2000) (Kang et al. 2000) , which are still health hazards [4]. However, Azbar et al. (2004) reported that complete dearomatization of dye is associated with AOP process [42]. The cleavage of azo bonds and formation of aromatic amines with the amines which are linked to the dyes are also reported by Janakar et al. (2006) [43].

The results of GC–MS analysis of the effluents treated by the Fenton–biological process showed a significant reduction in aromatic amines (Figure 6a). Decreasing in peaks implies the progress in the mineralization of RB5 and its metabolites. Therefore, dearomatization occurs in the biological stage. Our results demonstrated positive effects of inoculating bacterial consortium which was capable of dye biodegradation with a Fenton’s pretreatment step as well as the benefits of low time required for the biological process.

## Conclusions

Two stages, i.e. Fenton oxidation and anoxic biological treatment, were integrated with the purpose of reducing the treatment costs via less need for oxidant and catalyst and reduced sludge production. In the biological process alone, decolorization rate increased by increasing the initial dye concentration; the decolorization rate was 3.1 mg/L.h for an initial RB5 concentration of 500 mg/L. In the Fenton oxidation process alone, the optimum conditions to obtain the highest decolorization rate were: 0.5 mM Fe^2+^, 2.9 mM H_2_O_2_, pH 3, and a detention time of 15 min. After Fenton oxidation process, some aromatic amines intermediates were produced, suggesting that partial mineralization occurred in the process.

In the combined Fenton–biological process, lower doses of Fenton’s reagents were used to have the minimum negative effect of H_2_O_2_ residual on the biological stage. In this stage, the retention time reduced to half of that needed for biological treatment alone; significant reductions also occurred in the concentration of aromatic amines intermediates. Our results indicated that Fenton oxidation process combined with anoxic biological process is a cost–effective method for dye decolorization as well as for dearomatization of aromatic amines intermediates.

## Competing interests

The authors declare that they have no competing interests.

## Authors’ contributions

Pegah Bahmani, Roshanak Rezaei Kalantary, Ali Esrafili , Mitra Gholami and Ahmad Jonidi Jafari carried out the article with the title of: Evaluation of Fenton oxidation process coupled with biological treatment for the removal of reactive black 5 from aqueous solution, participated in the sequence alignment and drafted the manuscript. All authors read and approved the final manuscript.
